# Mutations in *KIAA1109*, *CACNA1C*, *BSN*, *AKAP13*, *CELSR2*, and *HELZ2* Are Associated With the Prognosis in Endometrial Cancer

**DOI:** 10.3389/fgene.2019.00909

**Published:** 2019-11-07

**Authors:** Zhiwei Qiao, Ying Jiang, Ling Wang, Lei Wang, Jing Jiang, Jingru Zhang

**Affiliations:** The Department of Gynaecology, Liaoning Cancer Hospital & Institute, Cancer Hospital of China Medical University, Shengyang, China

**Keywords:** endometrial cancer, bioinformatics analyses, mutation, overall survival time, biomarkers

## Abstract

Endometrial cancer (EC) is one of the most common gynecologic malignancies. Emerging studies had demonstrated the mutations in genes could serve as diagnostic or prognostic markers for human cancers. In this study, we screened mutated genes in EC and found that the mutations in *KIAA1109, CACNA1C, BSN, AKAP13, CELSR2,* and *HELZ2* were correlated to the overall survival time in patients with EC. Bioinformatics analysis showed *KIAA1109* was involved in regulating NIK/NF-kappaB signaling, *CACNA1C* was found to regulate cell migration and proliferation, *BSN* was found to regulate Wnt signaling pathway, *CELSR2* was involved in regulating cell–cell adhesion, nuclear import, and protein folding, *HELZ2* was found to regulate multiple immune related biological processes, and *AKAP13* was involved in regulating translation, mRNA nonsense-mediated decay, rRNA processing, translational initiation, and mRNA splicing *via* spliceosome. The findings provided a novel therapeutic strategy in patients with EC.

## Introduction

Endometrial cancer (EC) is one of the most common gynecologic malignancies (Attarha et al., 2011). Despite the prognosis of the early stage EC is good with a 5-year survival rate of 69–88% (Gottwald et al., 2010). However, the prognosis of metastatic EC remained very poor, with a median survival of 7–12 months. Therefore, there is an urgent need to identify novel biomarkers for the prognosis of EC. Moreover, the mechanisms underlying the progression of EC remained largely unclear.

With the development of next-generation sequencing, multiple EC related mutations were identified. Emerging studies had demonstrated the mutations in genes could serve as diagnostic or prognostic markers for human cancers. For example, McConechy et al. identified a series of mutations in *PTEN, CTNNB1, PIK3CA, ARID1A, ARID5B*, and *KRAS* were associated with EC (Mcconechy et al., 2012). The mutations in FGFR2 were associated with poor outcomes in endometrioid endometrial cancer (Jeske et al., 2017). The genetic alterations in CTCF could promote EC cell survival and alter cell polarity (Marshall et al., 2017). Jing et al. found that MUC16 mutations could improve patients’ prognosis by enhancing the infiltration of cytotoxic T lymphocytes in the EC microenvironment (Jing and Jing, 2014).

The present study identified prognosis related gene mutations in EC by analyzing TCGA databases (Collins, 2007). The mutations in 6 genes were correlated to the overall survival time in patients with EC. Bioinformatics analysis was used to predict the potential functions of these genes. The purpose of this study was to evaluate the impact of somatic tumor mutation on recurrence-free survival in this patient population.

## Materials and Methods

### Data Mining With cBioPortal and TCGA Database

In this study, we identified the gene mutations in EC using TCGA database (https://portal.gdc.cancer.gov/). All searches were performed according to cBioPortal’s online instructions (http://www.cbioportal.org/index.do) (Jianjiong et al., 2013). The survival analysis related to gene mutations was performed on the TCGA database (https://portal.gdc.cancer.gov/). 

### Co-Expression Network Analysis

In this study, the Pearson correlation coefficient was calculated according to the expression value between lncRNA–mRNA pair using cBioPortal’s online instructions (http://www.cbioportal.org/index.do). The top 500 co-expressing genes were selected as potential targets of mutated genes in EC.

### Bioinformatics Analysis

GO and KEGG pathway enrichment analysis were performed to determine the biological significance of DEGs, using the Database for Annotation, Visualization, and Integrated Discovery (Dennis et al., 2003) (DAVID; version 6.8; http://david.ncifcrf.gov/). 

### Patients’ Prognostic Analyses

Survival curves were depicted using the Kaplan-Meier method and compared with log-rank test. Cox proportional hazards regression analysis was used for univariate and multivariate analyses to explore the association of clinical features, gene mutational status, and patients’ prognosis. All the prognostic analyses were conducted by survival R package.

### Statistical Analysis

The two groups were compared using Student’s t‐test. Overall survival time analyses were estimated using the Kaplan-Meier product-limit estimator, and then a log-rank test was conducted to compare wildtype and mutation status. Overall survival was measured from the date of surgery to the date of last contact or death. Patients alive were censored at the date of last contact or clinic visit. Stata v14.2 (College Station, TX) was used to conduct statistical analysis.

## Results

### Screening of Mutated Genes in Endometrial Cancer

**Figure 1:**
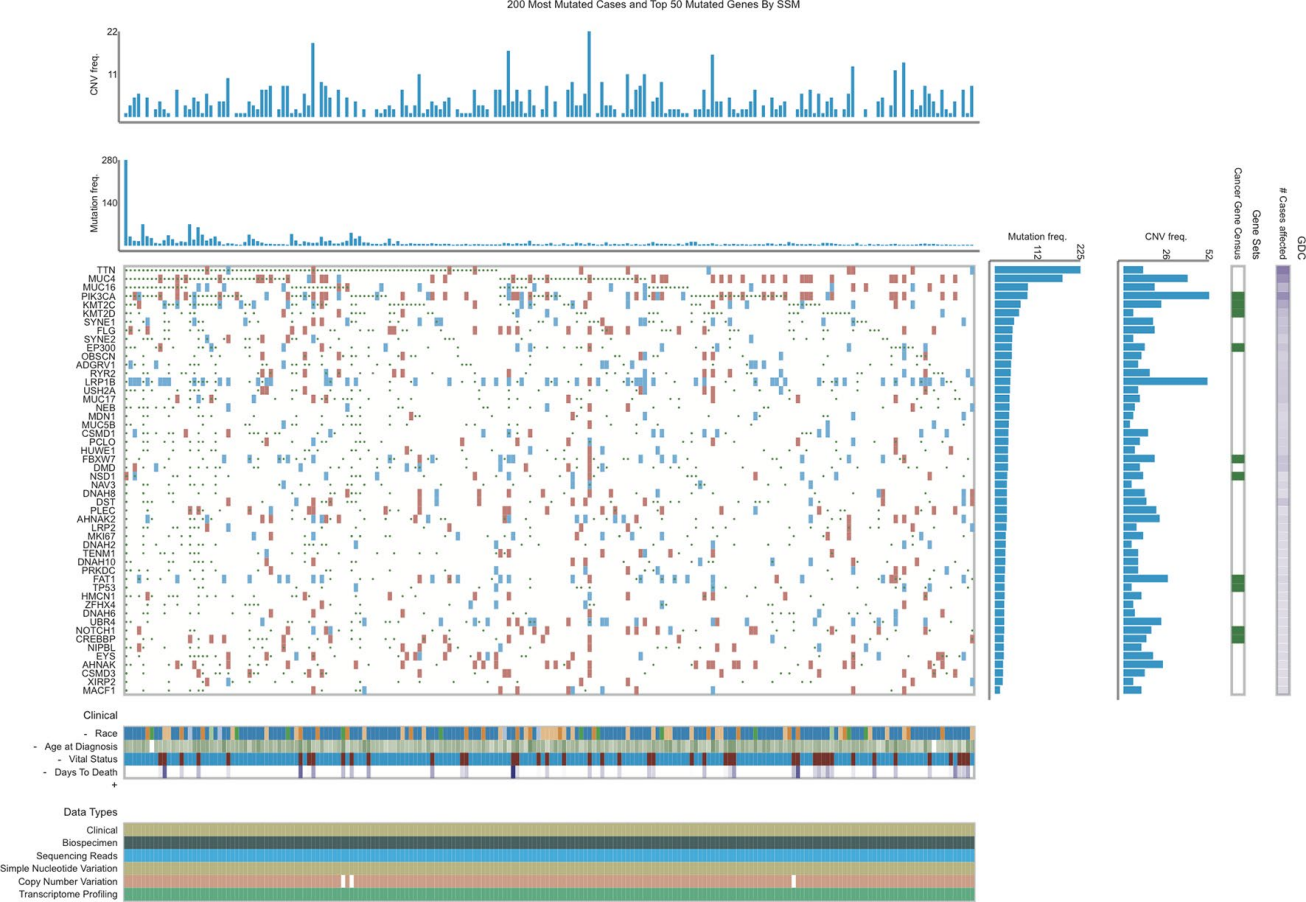
Identification of mutated genes in EC using TCGA database.

The present study analyzed TCGA database to identify mutated genes in EC. As shown in **Figure 1**, the top 50 mutated genes in EC included *TTN, MUC4, MUC16, PIK3CA, KMT2C, KMT2D, SYNE1, FLG, SYNE2, EP300, OBSCN, ADGRV1, RYR2, LRP1B, USH2A, MUC17, NEB, MDN1, MUC5B, CSMD1, PCLO, HUWE1, FBXW7, DMD, NSD1, NAV3, DNAH8, DST, PLEC, AHNAK2, LRP2, MKI67, DNAH2, TENM1, DNAH10, PRKDC, FAT1, TP53, HMCN1, ZFHX4, DNAH6, UBR4, NOTCH1, CREBBP, NIPBL, EYS, AHNAK, CSMD3, XIRP2,* and *MACF1*. Among these genes, *TTN*, *MUC4*, and *PIK3CA* are the most frequently mutated genes. The mutation rates in *TTN*, *MUC4*, and *PIK3CA* from the TCGA provisional data sets were 43.25% (125/289), 31.83% (92/289), and 29.41% (85/289), respectively.

### The Somatic Mutations of *KIAA1109, CACNA1C, BSN, AKAP13, CELSR2,* and *HELZ2* Were Correlated to Overall Survival Time in Patients With EC

**Figure 2:**
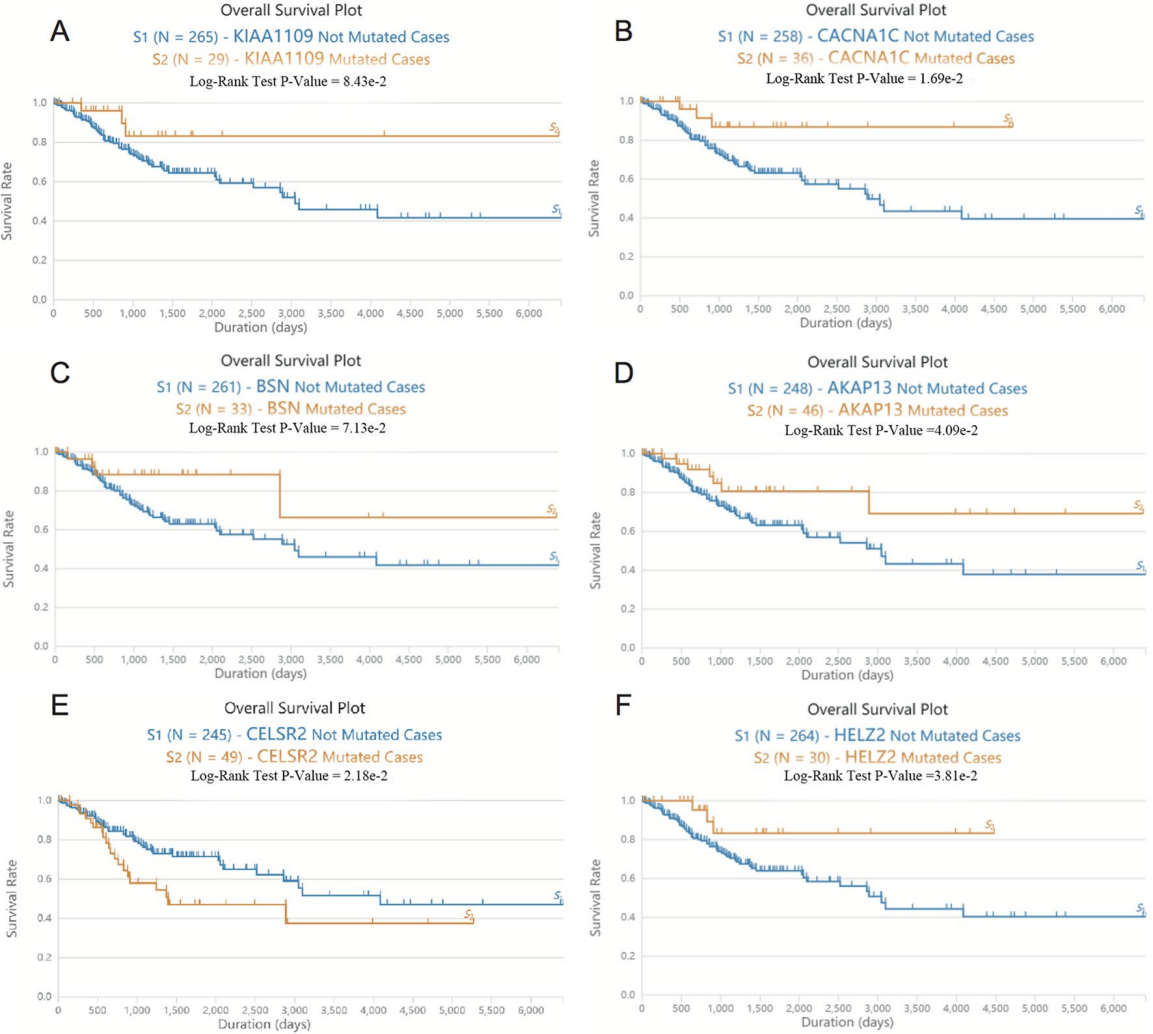
The somatic mutations of *KIAA1109, CACNA1C, BSN, AKAP13, CELSR2*, and *HELZ2* were correlated to overall survival time in patients with EC. **(A–F)** Log-rank test showed that mutations in *KIAA1109* **(A)**, *CACNA1C* **(B)**, *BSN* **(C)**, *AKAP13* **(D)**, *CELSR2* **(E)** and *HELZ2* **(F)** were associated with the overall survival time in EC patients.

Next, we screened somatic mutations associated with overall survival time in patients with EC. As shown in **Figure 2**, Log-rank test showed that mutations in *KIAA1109, CACNA1C, BSN, AKAP13,* and *HELZ2* were significantly associated with the longer overall survival time in EC patients, however, mutations in CELSR2 were significantly associated with the shorter overall survival time in EC patients.

### Mutation Profiles in *KIAA1109*, *CACNA1C*, *BSN*, *AKAP13*, *CELSR2*, and *HELZ2* in EC

**Figure 3:**
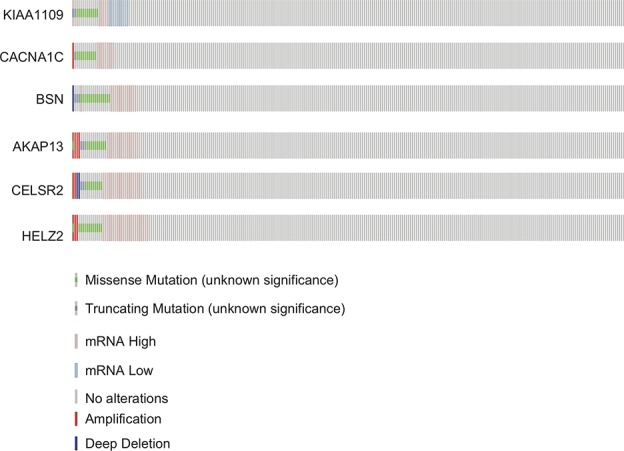
Mutation profiles of *KIAA1109*, *CACNA1C*, *BSN*, *AKAP13*, *CELSR2* and *HELZ2* found in EC.

**Figure 4:**
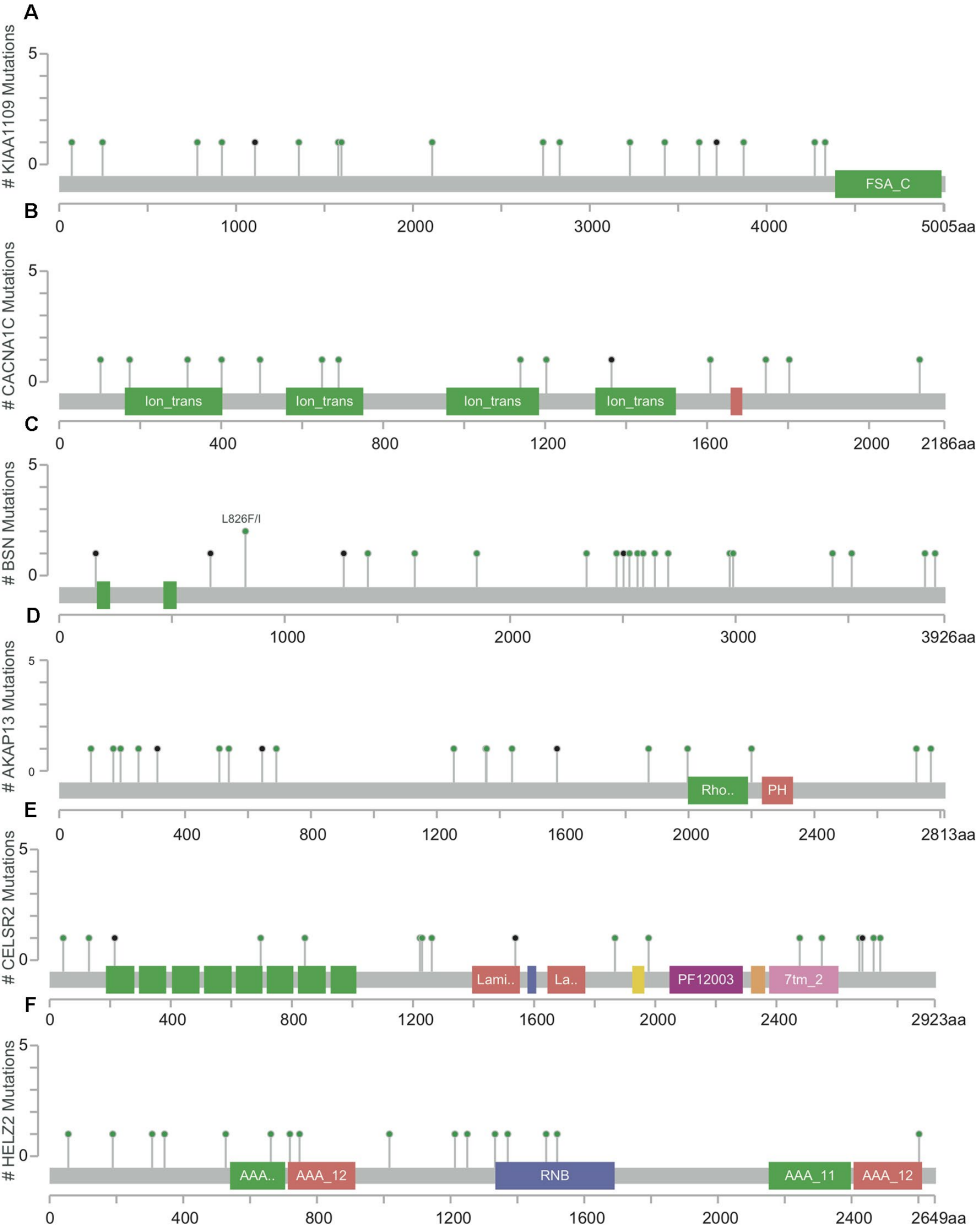
Detailed mutation maps of *KIAA1109* **(A)**, *CACNA1C* **(B)**, *BSN* **(C)**, *AKAP13* **(D)**, *CELSR2* **(E)** and *HELZ2* **(F)** found in patients with EC. Each dot above the protein molecule represents a mutation, which spreads across the entire encoded protein of these genes.

The mutation rates in *KIAA1109, CACNA1C, BSN, AKAP13, CELSR2,* and *HELZ2* from the TCGA provisional data sets were 6.92% (20/289), 7.27% (21/289), 7.96% (23/289), 7.61% (22/289), 6.92% (20/289), and 7.27% (21/289), respectively in **Figure 3**. A, majority of mutations identified were missense and nonsense resulting in amino acid, changes and a truncation of these proteins. However, there was no evidence of a mutational hotspot in *KIAA1109, CACNA1C, BSN, AKAP13, CELSR2,* and *HELZ2* in EC patients (**Figure 4**).

### The Effect of Mutations on mRNA Expressions of *KIAA1109, CACNA1C, BSN, AKAP13, CELSR2,* and *HELZ2* in EC Patients

**Figure 5:**
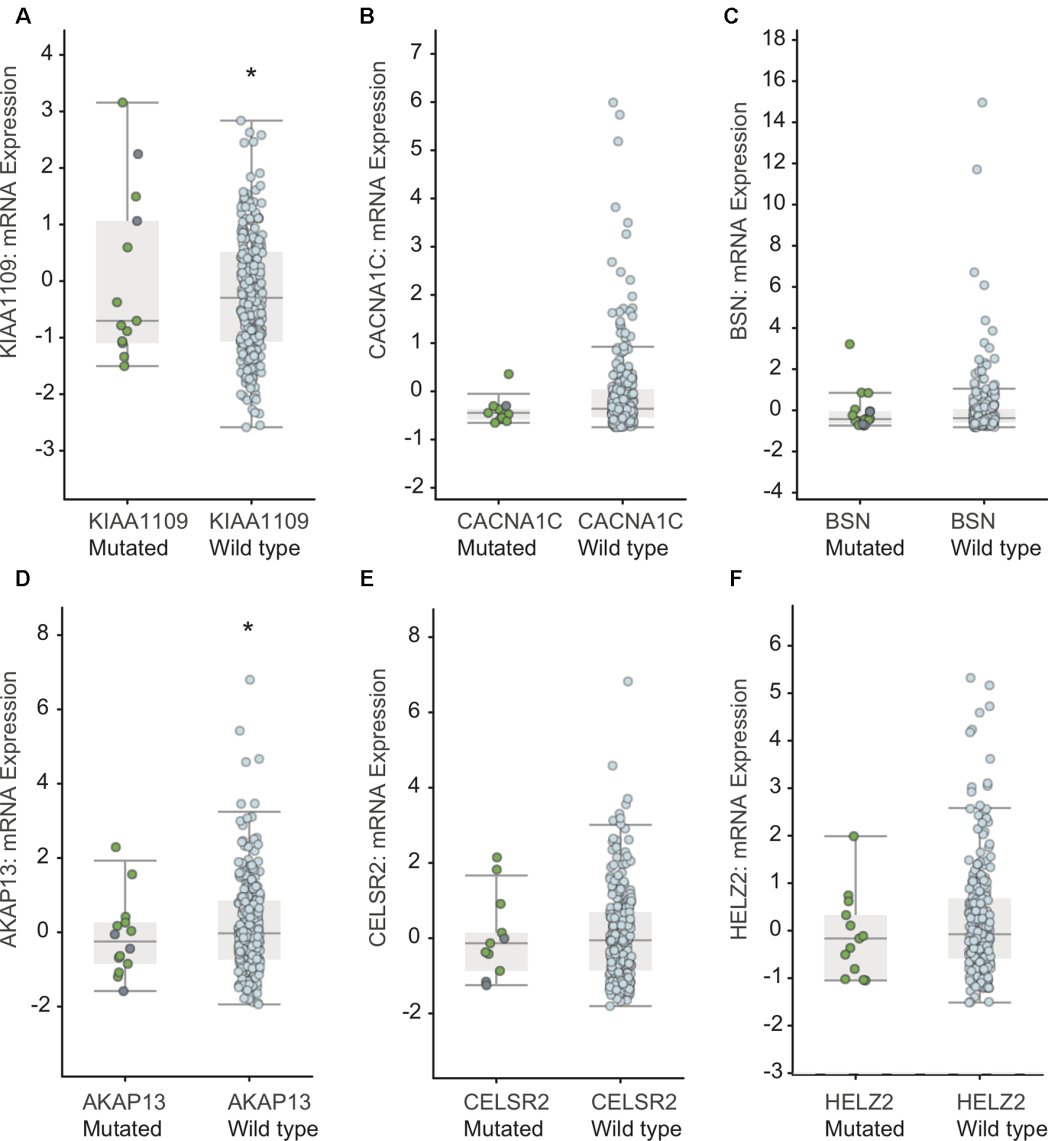
Association between mutations in *KIAA1109* **(A)**, *CACNA1C* **(B)**, *BSN* **(C)**, *AKAP13* **(D)**, *CELSR2* **(E)** and *HELZ2* **(F)** and mRNA levels in EC samples. * means p value < 0.05 between the two groups.

Furthermore, we detected the effect of mutations in *KIAA1109, CACNA1C, BSN, AKAP13, CELSR2,* and *HELZ2* on mRNA expression based on the RNA-Seq data. As shown in **Figure 5**, we found the mutations in *CACNA1C*, *BSN*, *CELSR2*, and *HELZ2* did not result in a significant alteration of their mRNA levels. However, we found that the mRNA levels in *KIAA1109* and *AKAP13* mutated EC samples were lower than that in *KIAA1109* and *AKAP13* wild type EC samples.

### Bioinformatics Analysis of *KIAA1109*, *CACNA1C*, *BSN*, *AKAP13*, *CELSR2*, and *HELZ2* in EC Patients

**Figure 6:**
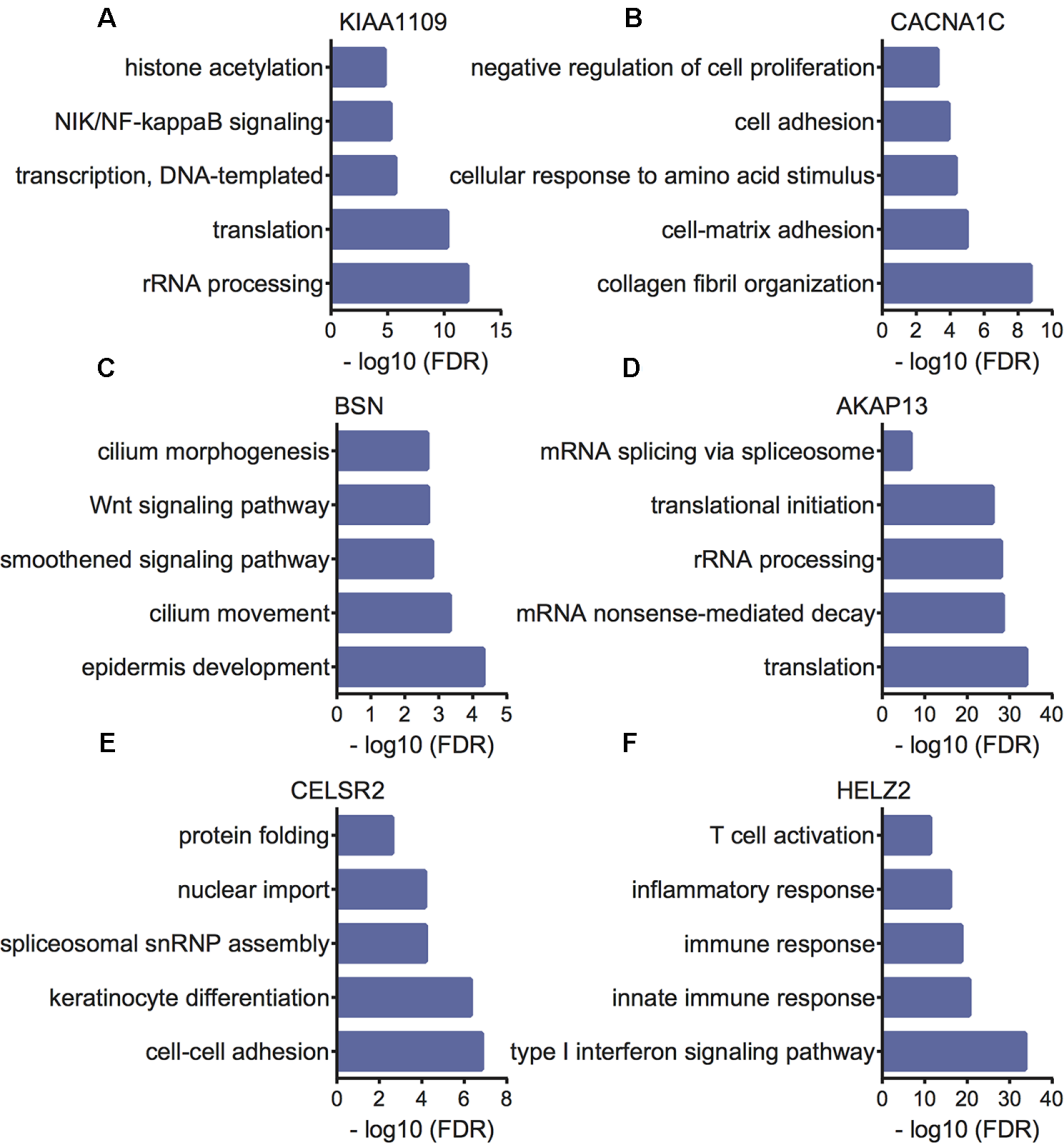
Bioinformatics analysis of *KIAA1109* **(A)**, *CACNA1C* **(B)**, *BSN* **(C)**, *AKAP13* **(D)**, *CELSR2* **(E)** and *HELZ2* **(F)** in EC samples.

Furthermore, we performed bioinformatics analysis to reveal the potential functions of *KIAA1109, CACNA1C, BSN, AKAP13, CELSR2,* and *HELZ2* using their co-expressing mRNAs in EC patients. The present study selected the top 500 correlated genes as the potential targets of *KIAA1109, CACNA1C, BSN, AKAP13, CELSR2,* and *HELZ2*. Bioinformatics analysis showed *KIAA1109* was involved in regulating rRNA processing, translation, transcription, NIK/NF-kappaB signaling, and histone acetylation. The results were shown in **Figure 6**. *CACNA1C* was involved in regulating collagen fibril organization, cell-matrix adhesion, cellular response to amino acid stimulus, cell adhesion, and negative regulation of cell proliferation. *BSN* was involved in regulating epidermis development, cilium movement, smoothened signaling pathway, Wnt signaling pathway, planar cell polarity pathway, and cilium morphogenesis. *AKAP13* was involved in regulating translation, mRNA nonsense-mediated decay, rRNA processing, translational initiation, and mRNA splicing *via* spliceosome. *CELSR2* was involved in regulating cell–cell adhesion, keratinocyte differentiation, spliceosomal snRNP assembly, nuclear import, and protein folding. *HELZ2* was involved in regulating type I interferon signaling pathway, innate immune response, immune response, inflammatory response, and T cell activation.

## Discussion

Endometrial cancer (EC) is one of the most common gynecologic malignancies. However, the mechanisms underlying EC progression remained unclear. Previous studies had showed the mutations in several genes were related to EC. For example, *MUC16* mutations improve EC prognosis through enhancing the infiltration of cytotoxic T lymphocytes. *PTEN* and *PIK3CA* mutations played crucial roles in grade 3 EC (Jing and Jing, 2014). The present study screened mutated genes in EC. Our results showed *TTN*, *MUC4*, and *PIK3CA* were the most frequently mutated genes in the EC, which was consistent with previous studies. Moreover, we identified the mutations in 6 genes were associated with the prognosis of EC. The results showed that mutations in *KIAA1109, CACNA1C, BSN, AKAP13,* and *HELZ2* were significantly associated with the longer overall survival time in EC patients. However, mutations in *CELSR2* were significantly associated with the shorter overall survival time in EC patients. These results suggested the important roles of these genes in the progression and prognosis of EC.

*KIAA1109*, located on the chromosome 4, was reported to be associated with susceptibility to celiac disease. Of note, 2 recent studies indicated *KIAA1109* was associated with the prognosis of human cancers. For example, Qing et al. reported mutations in *KIAA1109, DNAH5* and *KCNH7* were associated with poor survival of Chinese esophageal squamous cell carcinoma patients (Tao et al., 2017). Tindall et al. found genetic variation of *KIAA1109* might be associated with prostate cancer susceptibility in men with a family history of the disease (Tindall et al., 2010). *CACNA1C* gene encodes an alpha-1 subunit of a voltage-dependent calcium channel (Fayi et al., 2016). The mutations in *CACNA1C* were observed in various types of human diseases, such as ventricular fibrillation, and schizophrenia (Charles et al., 2007). Previous studies showed *CACNA1C* was down-regulated in multiple human cancers (Fastje et al., 2009), including brain tumors, kidney cancers and lung cancers, suggested its regulatory roles in cancer progression. BSN encoded a scaffolding protein involved in organizing the presynaptic cytoskeleton. BSN has been demonstrated to have chemo-preventive, antiproliferative, antifungal, and anti-carcinogenic activities. In addition, *BSN* has been reported to induce G1 phase arrest through increase of p21 and p27. In PCa, *BSN* was involved in regulating cell apoptosis in cancer cells (Xu et al., 2016). The dysregulation and mutation of *AKAP13* were found to be associated with the progression of colorectal cancer and breast cancer. Bentin et al. showed *AKAP13* is essential for the phosphorylation of ERαS305 (Toaldo et al., 2015), which leads to tamoxifen resistance in breast cancer. *HELZ2* encoded b a nuclear transcriptional co-activator for peroxisome proliferator activated receptor alpha (Jakobsson et al., 2010). However, its roles in human cancers remained largely unclear. *CELSR2* was found to be dysregulated in breast cancer (Jiang et al., 2018). However, the potential functions of *CELSR2* in EC remained unknown.

In the present study, we performed co-expression analysis to reveal the potential roles of these mutated genes in EC. The results showed *KIAA1109* was involved in regulating NIK/NF-kappaB signaling. Of note, NF-kappaB signaling had been demonstrated to be a key regulator in cancers. Suppressing of NF-kappaB signaling could inhibit cell growth and invasion in multiple cancers. For example, NF-κB suppresses apoptosis and promotes the proliferation of bladder cancer cells. A recent study showed liposomal curcumin targeting EC through the NF-κB Pathway. Bioinformatics analysis revealed *CACNA1C* played important roles in regulation of EC metastasis and proliferation. *BSN* was found to regulate Wnt signaling pathway. Mounting evidence has confirmed the activation of Wnt/β-catenin signaling was associated with multiple cancers, including EC. *AKAP13* was predicted as a RNA processing regulator. *CELSR2* was involved in regulating cell–cell adhesion, keratinocyte differentiation, spliceosomal snRNP assembly, nuclear import, and protein folding. *HELZ2* was involved in regulating type I interferon signaling pathway, innate immune response, immune response, inflammatory response, and T cell activation. These results suggested these mutated genes played important roles in EC tumorigenesis and progression.

Despite that bioinformatics analyses were conducted to predict the potential functions of these mutated genes in EC, several limitations still existed in this study. First, the mutated sites of these genes should be further validated in EC clinical samples using Sanger sequencing. Second, the molecular function of these key mutated genes in EC remained unclear. Therefore, gain or loss of function assays should be further conducted to investigate their important roles in EC.

In conclusion, we screened mutated genes in EC and found that the mutations in *KIAA1109, CACNA1C, BSN, AKAP13, CELSR2*, and *HELZ2* correlated with the overall survival time in patients with EC. Bioinformatics analysis showed *KIAA1109* was involved in regulating NIK/NF-kappaB signaling, *CACNA1C* was found to regulate cell migration and proliferation, *BSN* was found to regulate Wnt signaling pathway, *CELSR2* was involved in regulating cell-cell adhesion, nuclear import, and protein folding, and *HELZ2* was found to regulate multiple immune related biological processes. The findings provided a novel therapeutic strategy in patients with EC.

## Data Availability

All datasets analysed in this study can be found in the Genomic Data Commons Data Portal (https://portal.gdc.cancer.gov/). We screened the gene mutations in EC. We have downloaded these data from the database and the top 500 mutated genes in EC were listed in **Supplementary Table 1**.

## Author Contributions

JZ designed experiments; ZQ and YJ analyzed the data. All authors wrote and approved the manuscript.

## Funding

This work is supported by the Natural Science Foundation of Liaoning Province, China (Grant no. 20170540570).

## Conflict of Interest Statement

The authors declare that the research was conducted in the absence of any commercial or financial relationships that could be construed as a potential conflict of interest.

## Supplementary Material

Supplementary Table 1 The top 500 mutated genes in EC.
